# *De Novo* Designed Proteins from a Library of Artificial Sequences Function in *Escherichia Coli* and Enable Cell Growth

**DOI:** 10.1371/journal.pone.0015364

**Published:** 2011-01-04

**Authors:** Michael A. Fisher, Kara L. McKinley, Luke H. Bradley, Sara R. Viola, Michael H. Hecht

**Affiliations:** Departments of Chemistry and Molecular Biology, Princeton University, Princeton, New Jersey, United States of America; Center for Genomic Regulation, Spain

## Abstract

A central challenge of synthetic biology is to enable the growth of living systems using parts that are not derived from nature, but designed and synthesized in the laboratory. As an initial step toward achieving this goal, we probed the ability of a collection of >10^6^ *de novo* designed proteins to provide biological functions necessary to sustain cell growth. Our collection of proteins was drawn from a combinatorial library of 102-residue sequences, designed by binary patterning of polar and nonpolar residues to fold into stable 4-helix bundles. We probed the capacity of proteins from this library to function *in vivo* by testing their abilities to rescue 27 different knockout strains of *Escherichia coli,* each deleted for a conditionally essential gene. Four different strains – Δ*serB*, Δ*gltA*, Δ*ilvA*, and Δ*fes* – were rescued by specific sequences from our library. Further experiments demonstrated that a strain simultaneously deleted for all four genes was rescued by co-expression of four novel sequences. Thus, cells deleted for ∼0.1% of the *E. coli* genome (and ∼1% of the genes required for growth under nutrient-poor conditions) can be sustained by sequences designed *de novo.*

## Introduction

In 1906 Jacques Loeb suggested that the synthesis of life is a significant goal of biology [1]. A century later, the construction of natural genomes from off-the-shelf chemicals [2], [3] demonstrates significant progress toward achieving this goal. Until now, however, most advances in synthetic biology have relied on collections of parts – genes, proteins, and regulatory elements – derived from sequences that already exist in nature. Must the toolkit of life be so restricted? Natural sequences comprise only a miniscule fraction of the theoretical sequence space that is possible for genes and proteins. Indeed, a collection containing just a single molecule of every one of the 20^100^ possible 100-residue proteins would fill a volume larger than a mole of universes [4]. From this enormous potential for diversity, natural selection has operated over billions of years to yield a relatively small collection of sequences: Life is sustained by only ∼4,000 genes in *E. coli* and approximately 5-fold more in humans [5], [6]. These considerations might lead to the supposition that genes and proteins capable of sustaining life are somehow “special.” Is this true? Or might we find functional molecular parts in a collection of artificial sequences designed “from scratch” in the laboratory?

**Figure 1:**
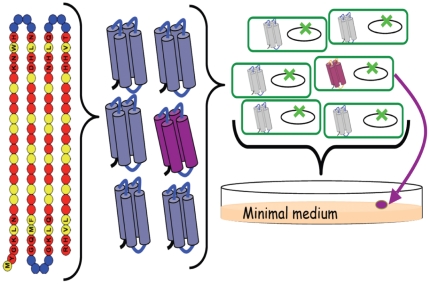
Design of a collection of novel proteins and rescue of *E. coli* auxotrophs. Binary pattern of polar (red) and nonpolar (yellow) residues designed to fold into 4-helix bundles (*7, 8*). Library construction is facilitated by the organization of the genetic code: Six polar residues (Lys, His, Glu, Gln, Asp, and Asn) are encoded by the degenerate codon VAN, and five nonpolar residues (Met, Leu, Ile, Val, and Phe) are encoded by the degenerate codon NTN. Combinatorial turn positions are encoded by the degenerate codon VRS, which encodes Gln, Glu, Asn, Asp, His, Lys, Arg, Ser, and Gly. (V = A, G, or C; R = A or G; N = A, G, C, or T). Circles with letters indicate fixed residues, and empty circles indicate combinatorially diverse positions. The theoretical diversity of this library is 5^22^×6^34^×8^12^ = 5×10^52^. The substantially smaller experimental library (1.5×10^6^ synthetic genes cloned on a high expression plasmid) was transformed into a strain of *E. coli* in which an endogenous gene had been deleted (green X). Colony formation on minimal media indicates a novel sequence (purple) enables cell growth under selective conditions.

To address these questions, we probed the ability of unevolved sequence space to encode functions that enable cell growth. We designed and constructed a collection of artificial genes encoding approximately 1.5×10^6^ novel amino acid sequences. Because folding into a stable 3-dimensional structure is a prerequisite for most biological functions, we did not construct this collection of proteins from random sequences. Instead, we used the binary code strategy for protein design, shown previously to facilitate the production of large combinatorial libraries of folded proteins [7], [8]. In brief, the binary code strategy posits that stably folded proteins can be encoded by specifying the sequence pattern of polar and nonpolar residues (the binary pattern) to coincide with the exposed and buried parts of a structure, respectively. For example, to design a helical bundle comprising α-helices with hydrophilic faces exposed to aqueous solvent and hydrophobic faces buried in the interior of a protein, the binary pattern of polar (***P***) and nonpolar (***N***) residues should be ***PNPPNNPPNPPNNP***, consistent with the structural repeat of 3.6 residues per alpha-helical turn [9], [10]. For the current studies, we used the binary code strategy to design and construct a library of sequences designed to fold into 102-residue 4-helix bundles. Our strategies for protein design and selecting biological function are summarized in Figure 1. Details describing the construction and biophysical characterization of the library are presented elsewhere [11], [12].

Here we demonstrate that novel proteins from this binary patterned library can rescue *E. coli* cells that lack certain natural proteins required for cell viability. The novel proteins are substantially less active, and may function by different mechanisms than the natural proteins they replace. Nonetheless, co-expression of several novel proteins rescues a strain in which multiple natural genes were deleted simultaneously. These findings show that an organism, which would otherwise not grow, can be sustained by macromolecules devised in the laboratory.

## Results and Discussion

### *De novo* designed sequences rescue single-gene knockout strains of *E. coli*

**Figure 2:**
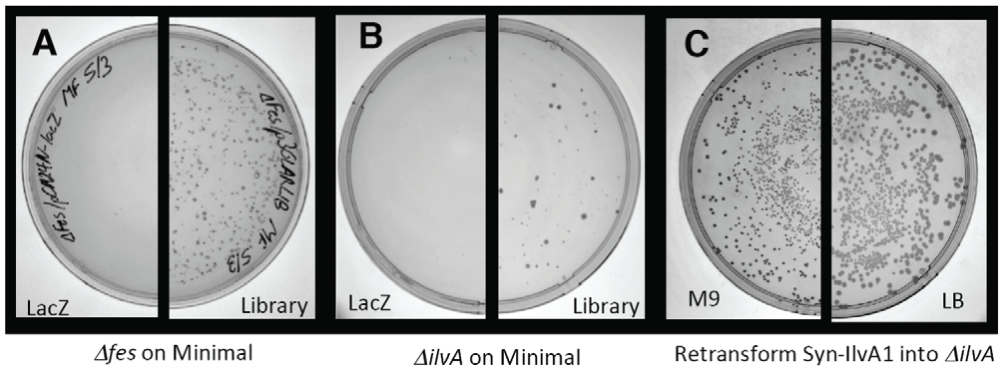
Rescue of *E. coli* auxotrophs by *de novo* proteins. (**A**) *E. coli* strain BW25113 Δ*fes* was transformed with a control plasmid overexpressing LacZ (left half), or with a library of plasmids encoding the collection of novel proteins (right half). Cells were plated on M9-glucose supplemented with 50 µM IPTG, 30 µg ml^−1^ kanamycin, and 34 µg ml^-1^ chloramphenicol. Cells transformed with the control plasmid fail to grow, whereas cells transformed with the library yield occasional colonies. (**B**) Similar results for Δ*ilvA*. (Similar results were also observed for Δ*serB* and Δ*gltA –* data not shown.) (**C**) Re-transformation of Δ*ilvA* cells with a single sequence, *syn-ilvA-1*. A similar number of colonies grow on M9-glucose (left) as on LB (right). Results for other sequences were similar to those shown in (C), and are summarized in Figure S1.

Previous work in our laboratory showed that several purified proteins from our binary patterned alpha-helical libraries can bind a cofactor and perform enzyme-like functions, including peroxidase, lipase, and esterase activities [13]. These findings led us to question whether proteins from this library might also provide biological functions that enable cell growth. To address this question, we tested the ability of binary patterned proteins to rescue strains of *E. coli* in which a conditionally essential function had been deleted. These strains were obtained from the Keio collection, which contains all the viable single-gene knockout strains of *E. coli* [5]. We tested 27 auxotrophs that grow on rich media, but fail to grow on minimal media (see legend for Table 1). Each auxotroph was transformed with a library of synthetic genes carried on the expression vector pCA24NMAF2 [14]. Typically, we obtained 5–10 million transformants, thereby ensuring reasonably deep coverage of the library of 1.5×10^6^ *de novo* sequences. As negative controls, each auxotroph was transformed with the empty vector or the same vector expressing beta-galactosidase. Transformed cells were spread either on LB (rich) or on M9-glucose (minimal) media supplemented with isopropyl-beta-D-thiogalactoside (IPTG) to induce expression, and the formation of colonies was monitored (Figures 1 & 2).

**Table 1:** Complementation frequency and time required for colony formation on selective media (M9-glucose).

	Complementation Frequency	Time to Grow (Days)
Δ*serB*	∼1/50,000	4
Δ*gltA*	∼1/1,000,000	4
Δ*ilvA*	∼1/3,000	3
Δ*fes*	∼1/10,000	2

Novel proteins capable of enabling cell growth were isolated for 4 strains. Complementation frequency was calculated by dividing the number of colonies on selective plates (rescued transformants) by the number of colonies on rich plates (total transformants). This apparent frequency was then adjusted to estimate the true complementation rate by correcting for any false positives that failed to restreak or retransform. The following additional strains from the Keio collection were tested, but were not rescued by sequences from the library: Δ*argB*, Δ*argE*, Δ*argH*, Δ*aroB*, Δ*aroC*, Δ*aroD*, Δ*aroE*, Δ*bioA*, Δ*cysG*, Δ*glnA*, Δ*hisA*, Δ*ilvC*, Δ*ilvD*, Δ*leuB*, Δ*leuC*, Δ*leuD*, Δ*pdxA*, Δ*ppc*, Δ*proB*, Δ*purF*, Δ*pyrF*, Δ*thrB*, and Δ*thrC*.

On rich media, all transformed cells grew, regardless of whether they received the control *lacZ* gene or a gene encoding a novel protein. As expected, on minimal media, auxotrophs transformed with the control plasmids failed to grow. (If the negative controls grew, the strain was considered ‘leaky’ and not used for further studies.) In most cases, auxotrophs transformed with the collection of novel sequences also failed to produce colonies, even after weeks of incubation. This indicates that proteins from the collection could not rescue these strains. However, for four auxotrophs – Δ*serB*, Δ*gltA*, Δ*ilvA*, and Δ*fes –* colonies formed on minimal plates after several days of incubation (Figures 2A & 2B). Growth of these cells under selective conditions suggests that a novel gene carried by the plasmid complements the deletion. The rates of complementation and the times required for colony formation are summarized in Table 1.

To confirm that the colonies observed on selective media resulted from the uptake of a novel gene and not from an adaptive mutation on the chromosome, colonies were isolated by restreaking, and plasmid DNA was purified; this DNA was then used to transform naïve cells of the same auxotroph, and the transformed cells were once again spread on both LB and minimal plates. Growth of approximately the same number of colonies on selective plates as on rich plates indicates that the observed phenotype (growth on minimal) transferred with the genotype (the plasmid carrying the novel gene). These results are shown in Figure 2C and Figure S1. To ensure that rescue was due to a *de novo* protein, and not some natural sequence that might have been picked up inadvertently during plasmid constructions, we isolated the ∼300 base pair fragment encoding the novel protein, recloned it into a new vector, and showed that the new clone also rescued the deletion strain.

**Figure 3:**
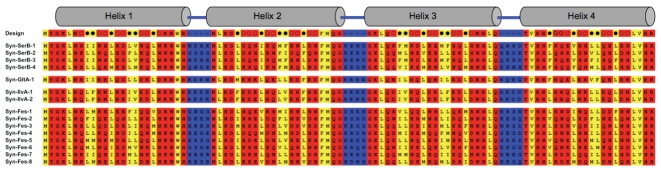
Designed amino acid sequences that enable growth of *E. coli* auxotrophs on selective media. Names of the novel (Syn) proteins and the auxotroph rescued are listed at left. The novel sequences follow the designed binary pattern of polar (red) and nonpolar (yellow) residues. The top line shows the template designed to encode four amphiphilic alpha-helices. Combinatorial positions are shown as empty circles (polar residues), filled circles (nonpolar residues), or hyphens (turn residues). Sequences were submitted to the European Nucleotide Archive and assigned accession numbers FR718891 - FR718908.

The binary patterned sequences that rescue the four auxotrophs are shown in Figure 3. (Additional sequences are shown in Figure S2.) The novel sequences are designated according to the auxotroph they rescue, followed by a number (e.g. Syn-Fes-1 is a synthetic sequence that rescues Δ*fes*.) For the Δ*gltA* strain, we isolated one novel protein that enabled cell growth. However, in the other cases, several sequences were isolated, suggesting that deletions of these genes are relatively easy to rescue. As shown by the color-coding in Figure 3, the sequences of the biologically active proteins conform to our binary pattern for the design of 4-helix bundles. (As is often true for sequences in combinatorial libraries, spurious mutations were observed occasionally, but these do not interfere with the overall binary pattern – see Figure S2). NCBI-BLAST searches indicate the sequences of the *de novo* proteins are unlike those of any known natural proteins [15].

**Figure 4:**
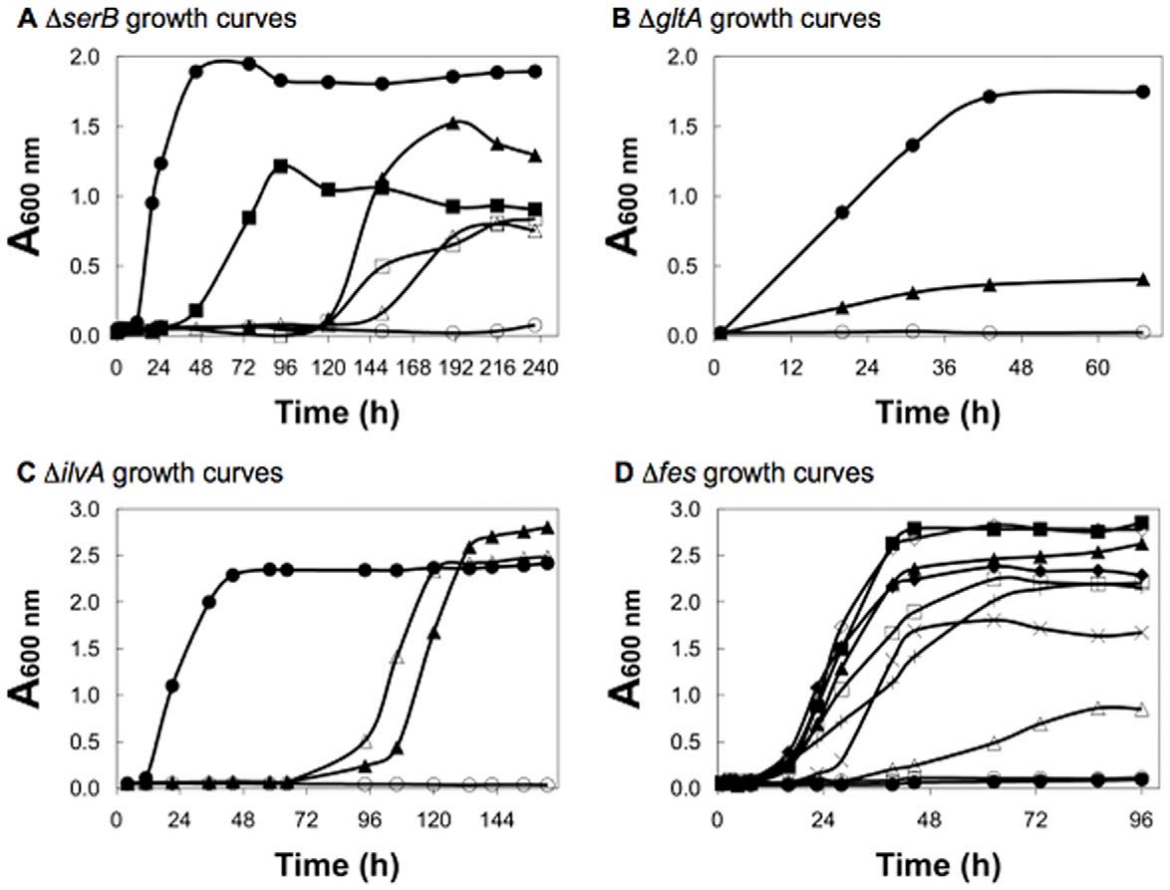
Growth of auxotrophic strains of *E. coli* in selective liquid media. In each panel, the bottom curve shows that the negative control expressing LacZ (open circles) does not support growth. In contrast, most of the positive controls (black circles) expressing the natural *E. coli* proteins grow well. (Fes is an exception because overexpression of *E. coli* Fes is toxic.) The designed proteins enable growth that is well above background. (**A**) Δ*serB*: LacZ, open circles; SerB, closed circles; Syn-SerB-1, closed triangles; Syn-SerB-2, open triangles; Syn-SerB-3, closed squares; and Syn-SerB-4, open squares. (**B**) Δ*gltA*: LacZ, open circles; GltA, closed circles; and Syn-GltA-1, closed triangles. (**C**) Δ*ilvA*: LacZ, open circles; IlvA, closed circles; Syn-IlvA-1, closed triangles; and Syn-IlvA-2, open triangles. (**D**) Δ*fes*: LacZ, open circles; Fes, closed circles; Syn-Fes-1, closed triangles; Syn-Fes-2, open triangles; Syn-Fes-3, closed squares; Syn-Fes-4, open squares; Syn-Fes-5, closed diamonds; Syn-Fes-6, open diamonds; Syn-Fes-7, ×; and Syn-Fes-8, +.

The ability of each *de novo* protein to sustain cell growth was measured in cultures grown in minimal M9-glucose liquid media. Growth rates were compared for deletion strains expressing a *de novo* protein versus the same strains expressing either the negative control (LacZ) or the corresponding natural protein on the same pCA24NMAF2 vector. As shown in Figure 4, the novel proteins enable cell growth under conditions where auxotrophs transformed with the control plasmid fail to grow at all. Cells relying on the *de novo* proteins grow significantly slower than those expressing the natural protein. (Fes is an exception because overexpression of *E. coli* Fes is toxic [14, Genobase ORF JW0576 - http://ecoli.naist.jp/GB6/info.jsp?id=JW0576].) It is not surprising that the unevolved novel sequences function at substantially lower levels than natural sequences selected by billions of years of evolution. Indeed, the relatively slow rates of cell growth enabled by these first-generation *de novo* sequences suggest that selections for faster growth might facilitate the evolution of more active proteins. Such experiments will provide an opportunity to test whether *de novo* progenitor sequences might lead to novel evolutionary trajectories.

The results shown in Figures 2 and 4 demonstrate that the *de novo* sequences enable cell growth; however, viability *per se* does not indicate that the novel proteins provide the same biochemical activities as the deleted natural proteins. Therefore, we devised a series of experiments to probe the functions of the *de novo* proteins.

### Biological functions of the *de novo* proteins

The natural genes deleted in these four auxotrophs encode a range of functions (9). These are summarized below and depicted graphically in Figure S3.

- *serB* encodes phosphoserine phosphatase, responsible for the final step in serine biosynthesis: phosphoserine → serine + phosphate.
- *gltA* encodes citrate synthase, which catalyzes an early step in glutamate biosynthesis: oxaloacetate + acetyl-coA → citrate → cis-aconitate → isocitrate → alpha-ketoglutarate → glutamate.
- *ilvA* encodes biosynthetic threonine deaminase, which catalyzes the first step in the production of isoleucine from threonine: threonine → 2-ketobutyrate + ammonia → → → isoleucine.
- *fes* encodes enterobactin esterase, which cleaves the iron-bound enterobactin siderophore, thereby enabling cells to acquire iron in iron-limited environments [16].

In principle, the novel proteins could rescue these auxotrophs either by providing the same function as the deleted enzyme, or by one of the three alternative mechanisms described below:

First, we considered the possibility that an auxotroph in a biosynthetic pathway might be rescued by a *de novo* sequence that produces the end product via a novel bypass pathway. Although it seems unlikely that small, unevolved proteins could catalyze novel biosynthetic pathways, we nonetheless tested this possibility. If the novel sequences that rescue Δ*serB,* Δ*gltA* or Δ*ilvA* encode bypass pathways for the synthesis of serine, glutamate, and isoleucine, respectively, then these *de novo* sequences would also rescue cells deleted for enzymes that function at other steps in the these biosynthetic pathways. (Pathways are shown in Figure S3). This possibility was tested by the following three experiments:

- *De novo* sequences that rescued Δ*serB* were transformed into Δ*serC* cells, which are deleted for phosphoserine aminotransferase. This enzyme converts phosphohydroxypyruvate to phosphoserine in the step prior to that catalyzed by the *serB* encoded enzyme. (Because the *serC* gene product is also involved in the biosynthesis of pyridoxine [9], the selective media for this experiment was supplemented with pyridoxine.)
- The sequence that rescued Δ*gltA* was transformed into Δ*icd* cells. This strain is also a glutamate auxotroph because it cannot catalyze the conversion of isocitrate to alpha-ketoglutarate, the direct precursor of glutamate.
- The sequences that rescued Δ*ilvA* were transformed into Δ*ilvC* and Δ*ilvD* cells, which are deleted for isomeroreductase and dihydroxyacid dehydratase, respectively. Both these enzymes function downstream of *ilvA* in the biosynthesis of isoleucine. (Because the *ilvC* and *ilvD* gene products are also involved in valine biosynthesis [9], the selective media for these experiments was supplemented with valine.)

In all cases, the *de novo* sequences did *not* rescue *E. coli* cells deleted for functions at other steps in the biosynthetic pathways. This demonstrates that the novel proteins do *not* enable bypass pathways for the synthesis of their respective end products.

Second, we considered the possibility that our *de novo* sequences might rescue the auxotrophs by altering the expression or activity of an endogenous *E. coli* protein. To assess this possibility, we relied on an exhaustive screen by Patrick *et al.*, who identified natural genes whose overexpression can rescue the deletion of noncognate genes in *E. coli* (i.e. ‘multicopy suppressors’) [17]. By screening the complete set of overexpressed *E. coli* ORFs, they found the following multicopy suppressors of our four strains: Δ*ilvA* can be rescued by overexpression of *tdcB* [18], or *emrD*; Δ*serB* can be rescued by overexpression of *gph, hisB,* or *ytjC*; and Δ*fes* can be rescued by overexpression of *thiL* or *setB*. Δ*gltA* was not rescued by overexpression of any *E. coli* genes. To probe whether our novel sequences rely on these *E. coli* proteins for rescue, we tested whether our proteins rescue the following double deletion strains: Δ*ilvA*Δ*tdcB,* Δ*ilvA*Δ*emrD,* Δ*serB*Δ*gph,* Δ*serB*Δ*ytjC,* and Δ*fes*Δ*setB*. Transformation with the appropriate novel sequences showed that all five of these double knockouts were rescued by all of the corresponding artificial sequences listed in Figure 3. The ability of our novel sequences to rescue these double deletions shows that the artificial proteins do *not* function by altering the expression or activity of *E. coli* proteins known previously to enable rescue.

The double mutant Δ*fes*Δ*thiL* cannot be constructed because deletion of *thiL* is lethal. The Δ*serB*Δ*hisB* strain was constructed, but since Δ*hisB* is itself an auxotroph, our novel sequences did not rescue this strain (successful rescue would require the unlikely occurrence of a single *de novo* protein replacing the activities of two conditionally essential genes.) Therefore, dependence on *thiL* and *hisB* could not be assessed. In addition, it remains possible that some other *E. coli* protein, which escaped selection by Patrick *et al*., could act as a partner in the rescue mediated by our *de novo* sequences. With the exception of these caveats, our results demonstrate that the known multicopy suppressors are not required for the biological activities of our *de novo* proteins.

Third, we considered the possibility that the *de novo* proteins might rescue the deletion strains by a mechanism that does not depend on the specific sequences (Figure 3), but instead involves global alterations in metabolism that are induced by the mere expression of foreign genes. For example, although our proteins were designed to fold into alpha-helical bundles, we considered the possibility that sequences isolated by our selections might be unfolded, and thereby induce a cellular stress response. To assess folding, we purified several proteins and measured their circular dichroism spectra. These spectra (shown in Figure S4) demonstrate the structures are predominantly alpha-helical, and similar to the spectra of designed 4-helix bundles solved previously by NMR [19], [20]. Thus, rescue is not caused by unfolded sequences inducing a stress response.

We also note that if a generic stress response were responsible for rescue, one would expect all the Syn proteins to rescue all the deletions. This is not the case. Syn-Fes does not rescue Δ*gltA*, Syn-SerB does not rescue Δ*ilvA*, and so on. Thus, specific *de novo* sequences mediate the rescue of specific chromosomal deletions.

To demonstrate explicitly that rescue depends on a specific sequence, rather than a generic cellular response to the expression of foreign genes, we mutated one of the *de novo* proteins. The design of this mutant was based on an analysis of the common features among the *de novo* sequences that rescued Δ*ilvA*. Alignment of the Syn-IlvA sequences revealed two conserved polar residues: Glu36 and Lys42. We constructed the Lys42»Ala mutation in Syn-IlvA-1, and found that although this protein was expressed at the same level as the parental sequence, it fails to rescue Δ*ilvA.* (These results are shown in Figure S5) Thus, rescue is not simply due to expression of an exogenous gene; it is mediated by sequence-specific features of Syn-IlvA-1.

Fes differs from the other deletions because it is not involved in a biosynthetic pathway. Fes functions in iron acquisition. *E. coli* secretes the enterobactin siderophore (MW, 670 Da) into the media, where it binds iron, and is transported back into the cell. Because the affinity of enterobactin for iron is extremely high (K_D_≈10^−35^ M), release of the metal requires degradation of the siderophore. This is catalyzed by the Fes protein, enterobactin esterase [16]. The impact of the *de novo syn-fes* sequences on iron acquisition is dramatic: Elemental analysis shows that cells expressing the Syn-Fes proteins accumulate 6- to 10-fold more iron than control cells (Figure S6). In principle, the *de novo* proteins could rescue Δ*fes* either by functioning as an esterase or by some alternative mechanism. For example, the *de novo* proteins could enable iron acquisition by being transported into the media, binding iron with high affinity, being re-transported back into the cell, and then releasing the tightly bound iron intracellularly. Although possible, this alternative mechanism seems unlikely for novel proteins that occur so frequently in a semi-random library.

Irrespective of whether the *de novo* proteins rescue auxotrophs by providing the same function as the deleted enzyme or by some alternative mechanism, we expected the artificial sequences – which were neither selected by evolution (*in vivo* or *in vitro*), nor explicitly designed for enzymatic activity – to have far lower levels of activity than naturally evolved sequences. To estimate this level of activity *in vivo*, we compared the growth rates of deletion strains expressing a high level (400X) of Syn-IlvA-1 to the same strain expressing a low level (1X) of the natural protein, encoded by IlvA. These experiments showed that even when the *de novo* protein is expressed at ∼400-fold higher levels than the natural protein, cells grow much more slowly. (Growth rates are shown in Figure S7.) As expected, the *de novo* protein exhibits a very low level of biological activity.

Activity levels that are barely sufficient to sustain slow cell growth may not be detectable *in vitro*. Indeed, others have reported that although overexpression of one *E. coli* protein can sometimes rescue the deletion of another protein *in vivo*, these ‘moonlighting’ activities can be so low that they cannot be detected *in vitro* [21], [22]. Despite these concerns, we attempted to assay biochemical activities *in vitro* using both cell lysates and purified proteins. Lysates are easy to prepare and are more likely to contain molecular partners (proteins, cofactors, or metals) present *in vivo*. The disadvantage of lysates, however, is that background activities may obscure the low-level activities of the *de novo* proteins. We assayed for phosphoserine phosphatase, enterobactin esterase, threonine deaminase, and citrate synthase activities in lysates from cells expressing the respective *de novo* proteins. In several cases, activity was observed, however, lysates from control cells also showed low levels of activity. This was not surprising, particularly for the Fes and SerB activities, since previous work in our laboratory showed that *E. coli* lysates contain nonspecific esterases and phosphatases [13].

We also purified several of the *de novo* proteins. (To avoid contamination by the natural enzyme, purifications were from strains deleted for the natural gene.) We tested these purified proteins for the enzymatic activities deleted in the respective auxotrophs, but were unable to detect activity that was reproducibly above the controls. There are several reasons why such experiments might not demonstrate activity: (i) As noted above, the novel proteins have extremely low levels of activity. (ii) The *de novo* proteins may require cofactors. We do not know which cofactors might be required, as the novel proteins may use different cofactors than the natural enzymes. (iii) The novel proteins may function by partnering with an *E. coli* protein. For example, the specificity of an endogenous hydrolase might be altered by binding one of our helical bundles. If this were the case, activity would not be observed in preparations containing only the purified *de novo* protein. (iv) The Syn protein may function by a different mechanism than the deleted protein, and novel activities would not be detected in experiments designed to assay the natural enzyme.

While we do not yet know the precise mechanisms by which the novel proteins rescue these four deletions in *E. coli,* we have ruled out several alternative mechanisms including (i) bypass pathways, (ii) activation of known endogenous suppressors, and (iii) induction of a generic stress response (see above). The slow growth rates of cells relying on the novel proteins for survival indicate that regardless of the actual mechanism, the *de novo* proteins have very low levels of activity — as expected for sequences that were neither designed nor evolved for function. Irrespective of whether the *de novo* proteins catalyze the same biochemical reaction as the deleted natural protein, or function by some alternative mechanism (and whether they act directly or through interactions with endogenous proteins), elucidating the molecular basis of auxotroph rescue will enhance our understanding of the minimal functions necessary for cell growth.

### Replacement of 0.1% of the *E. coli* genome

**Figure 5:**
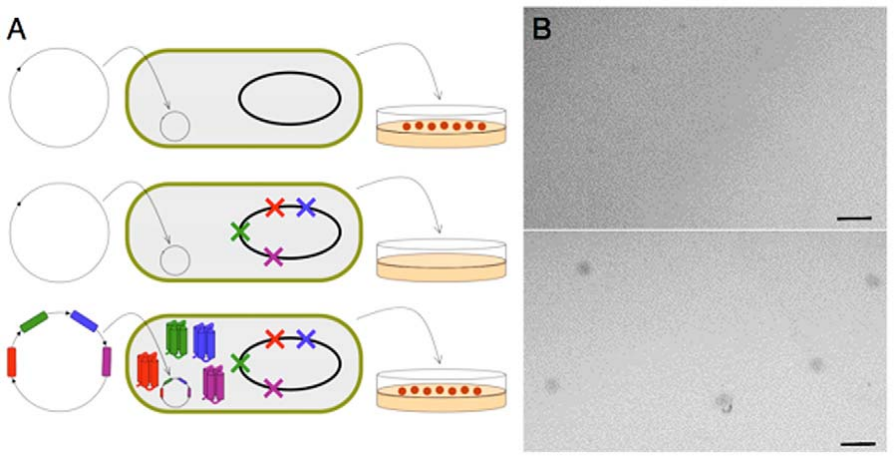
Rescue of a quadruple knockout *E. coli* by co-expression of 4 *de novo* proteins. (**A**) Schematic of knockout rescue. Top: Wild-type *E. coli* transformed with empty vector forms colonies on M9/glucose minimal media. Middle: QUAD *E. coli* transformed with empty vector does not form colonies on minimal media. Bottom: QUAD transformed with a vector co-expressing four *de novo* sequences forms colonies on minimal media. (**B**) Colonies are not observed when QUAD is transformed with empty vector (top panel), but are observed when transformed with a vector expressing four artificial genes (bottom panel). Scale bar = 1 mm.

We showed above that artificial genes enable cell viability. Next, to assess whether portions of a genome can be replaced by genetic information devised in the laboratory, we probed the ability of several novel sequences to rescue the deletion of several genes simultaneously. A quadruple knockout strain – Δ*fes* Δ*gltA* Δ*ilvA* Δ*serB* – was constructed and designated QUAD. This strain has severe growth defects: While the parent strain forms colonies after overnight incubation on either nutrient-rich or nutrient-poor media, QUAD requires almost two days to form colonies on rich media, and as expected, does not form colonies on minimal media. To assess the ability of artificial genes to substitute simultaneously for the deleted functions, we transformed QUAD with derivatives of the pQLinkN vector [23] co-expressing four artificial genes shown above to rescue the four single-gene knockouts. The sequences used for this experiment were *syn-fes-2*, *syn-gltA-1, syn-ilvA-1*, and *syn-serB-2* (Figure 3). Transformed cells were plated on minimal media. As a control, we transformed QUAD with empty vector; no colonies were observed for the control. However, as shown in Figure 5, transformation with the vector expressing all four artificial genes produced colonies. As discussed above, the individual Syn proteins are considerably less active than the deleted natural proteins. Therefore, it is not surprising that the simultaneous rescue of four deletions by four artificial proteins produced small colonies that required 13 days to appear. Nonetheless, the rescue of QUAD demonstrates that novel sequences bearing no similarity to naturally evolved proteins can compensate for deletion of a portion of the *E. coli* chromosome.

The *E. coli* genome contains approximately 4,000 genes [5]. Of these, only 290 are essential for survival under all conditions [24]. An additional 107 are required for growth in the nutrient-poor media provided by M9 salts with glucose [17]. Thus, only ∼400 genes are essential for growth in minimal media. Our results demonstrate that at least 1% of the *E. coli* genome that is absolutely required for growth under minimal conditions – and 0.1% of the entire *E. coli* genome – can be replaced by artificial genes designed *de novo.*

### Conclusion

We have demonstrated that sequences designed *de novo* can provide functions necessary to sustain the growth of living cells. It should be emphasized that these macromolecules were isolated with relatively high frequency from a collection of sequences that were designed to adopt a stable globular fold [7], [8], [19], [20], [25], but were not explicitly designed for function.

Both in terms of linear sequence and 3-dimensional structure, the novel proteins differ substantially from the natural proteins they replace: The binary patterned sequences in Figure 3 show no significant sequence similarity to any known proteins. At the structural level, the 102-residue 4-helix bundles specified by our design are much smaller and simpler than the structures of the four natural proteins deleted in the auxotrophs. (Structures are shown in Figure S8). Thus, although natural proteomes include diverse structures and topologies, our studies show that in some cases, cell growth can be sustained by much simpler structures.

The field of synthetic biology — and the related field of astrobiology — aim to go beyond questions about “what already exists” and attempt to probe “what might be possible?” The results described here suggest that the toolkit for synthetic biology need not be limited to genes and proteins that already exist in nature. We tested 27 deletion strains using a library of 1.5 million sequences designed to fold into 4-helix bundles. From these, we found 18 artificial sequences (Figure 3 and Figure S2) that compensate for the deletion of 4 different genes, and provide biological functions enabling cell growth. Extension of this work to other deletion strains, using larger libraries, and designs that encode a greater diversity of protein structures can be expected to increase substantially the number of life-supporting functions that can be provided by genes designed *de novo*. The results described here showing that sequences designed in the laboratory can replace portions of the *E. coli* genome can therefore be viewed as an initial step toward the construction of artificial genomes.

## Materials and Methods

### Construction of a library of *de novo* sequences

The design, construction, and characterization of a library of artificial genes encoding binary-patterned *de novo* proteins are reported elsewhere [11], [12]. The library of gene inserts was digested with NdeI and BsrGI, as was plasmid pCA24NMAF2. Double-digested plasmid backbone was gel purified, and the library insert and vector backbone were ligated. Ligation products were transformed into electrocompetent XL1-blue (Stratagene) or MDS42recA (Scarab Genomics, LLC). Colonies were harvested by scraping from LB plates supplemented with 34 µg ml^-1^ chloramphenicol (cam), and plasmid DNA was purified from the resulting cell suspension. Aliquots from this library of plasmids were used to transform the auxotrophs. The size of this library—approximately 1.5×10^6^ different sequences—was estimated by plating serial dilutions of the transformations of the initial ligation products on LB-cam [12].

### Isolation of *de novo* sequences that rescue auxotrophs

The collection of plasmids encoding our library of novel sequences, and two negative control plasmids, pCA24N-*lacZ* and pCA24NMAF2 (empty vector), were used to transform 27 *E. coli* auxotrophs. These were chosen from Keio strains that grow on LB, but not on M9-glucose. Following electroporation, cells were outgrown at 37°C, harvested by centrifugation, washed and resuspended in chilled M9 salts, and spread on both LB supplemented with 30 µg ml^−1^ kanamycin (kan), 34 µg ml^−1^ cam; and on M9-glucose supplemented with 50 µM IPTG, 30 µg ml^−1^ kan, 34 µg ml^−1^ cam. After several days at 33°C, plates were scored for number of colonies. In addition, a 1∶100 dilution of library-transformed cells was spread onto LB-cam plates to measure transformation frequency under conditions that were non-selective for auxotroph growth.

### Growth assays

Single colonies were inoculated into LB (30 µg ml^−1^ kan, 170 µg ml^−1^ cam). Following overnight growth at 37°C, 1 ml of each culture was harvested by centrifugation at 4°C. Cell pellets were washed and resuspended in 1 ml chilled M9 salts, and the resulting cell suspensions were diluted 100-fold into M9-glucose (50 µM IPTG, 30 µg ml^−1^ kan, 34 µg ml^−1^ cam). Cultures were grown at 33°C and assayed by measuring absorbance at 600 nm.

Additional methods are described in Supporting Information Text S1.

## Supporting Information

Text S1 Supplementary materials and methods.
(DOC)

Figure S1 Reconfirmation of hits. Colony counts for re-transformation of Δ*ilvA* cells with several different hits. The histogram summarizes 13 experiments of the type shown in figure 2C. The number of colonies on M9-glucose minimal media (dark blue) is similar to the number on LB rich media (light blue).
(TIFF)

Figure S2 De novo sequences with spurious mutations and corresponding Δ*ilvA* growth curves. (A) Novel sequences with spurious mutations. Red indicates polar residues, yellow indicates nonpolar residues, and turns are in blue. Syn-IlvA-3 and Syn-IlvA-4 are 105-residue proteins differing by a single residue at position 28. Both exhibit a 4 amino acid insertion into helix 1, and a 1 amino acid deletion from helix 4. Syn-IlvA-5 is a 106-residue protein, with an 11 amino acid insertion into the second helix and a 7 amino acid deletion from helix 4. These spurious mutations occur at the DNA level and are due either to errors in oligonucleotide synthesis or rare instances of the mis-assembly of library insert building blocks. As long as such spurious mutations do not affect frame or introduce stop codons, the mutated segments survive preselection, and as shown by our results, can contribute to the synthesis of productive sequences. (B) Growth curves for the Δ*ilvA* strain expressing designed artificial proteins with spurious mutations. The bottom curve shows that the negative control expressing LacZ (open circles) does not enable growth. In contrast, the positive control expressing IlvA (closed circles) grows well. Like the novel sequences that adhere precisely to the design, the designed proteins with spurious mutations enable growth that is well above background. Growth curves correspond to the sequences in **A** as follows: Syn-IlvA-3, closed squares; Syn-IlvA-4, open squares; and Syn-IlvA-5, closed diamonds.
(TIFF)

Figure S3 Reactions and pathways of the deleted proteins. (A) *serB* encodes phosphoserine phosphatase, responsible for the final step in serine biosynthesis. (B) *ilvA* encodes biosynthetic threonine deaminase, which catalyzes the first step in the production of isoleucine from threonine. (C) *fes* encodes enterobactin esterase, which cleaves iron-bound enterobactin. (D) *gltA* encodes citrate synthase, which catalyzes an early step in glutamate biosynthesis.
(TIFF)

Figure S4 Circular dichroism spectra. CD spectra demonstrate that purified *de novo* proteins fold into alpha-helical structures. Cultures of *E. coli* were grown to an OD600 of approximately 0.6 and induced with IPTG. Protein was extracted from the cells by repeated cycles of freezing and thawing. Purification of the Syn proteins to 95% (coommaise-stained SDS-PAGE) was achieved by cation exchange chromatography. Proteins were dialyzed into phosphate buffer for CD analysis.
(TIFF)

Figure S5 Mutation of Lys42 to Ala destroys the ability of a synthetic protein to rescue Δ*ilvA*. (A) Sequence alignment of the six synthetic proteins that rescue Δ*ilvA.* Two charged residues are conserved, E and K (red arrows). Green lines mark regions that are conserved in the designed template. (B) Transformation of Δ*ilvA* cells with Syn-IlvA-1 and Syn-IlvA-1-K42A. Cells expressing Syn-IlvA-1 grow on M9-glucose, but cells expressing Syn-IlvA-1-K42A do not. (C) SDS-PAGE of protein levels after induction with IPTG. Levels of Syn-IlvA-1-K42A are approximately equivalent to those of Syn-IlvA-1. Red arrow marks the band corresponding to the synthetic protein.
(TIFF)

Figure S6 Assay for iron using inductively coupled plasma (ICP) spectrometry. The Δ*fes* strain was transformed with a plasmid directing the high level expression of either LacZ or one of the artificial sequences that rescued the *fes* deletion. Cells were grown in LB, harvested, and tested for iron accumulation. The artificial proteins enable the accumulation of 6-10 fold more iron than the control.
(TIFF)

Figure S7 De novo proteins are considerably less active than the natural proteins they replace. (A) Growth on minimal media of Δ*ilvA* cells expressing different sequences. Cells expressing the natural IlvA protein grow well either in the presence (Blue) or absence (Red) of IPTG. In contrast, cells expressing Syn-IlvA-1 grow significantly more slowly, even in the presence of IPTG (Green). [These cells do not grow in the absence of IPTG.] Control cells expressing LacZ fail to grow even in the presence of IPTG (Purple). All proteins were expressed off the pCA24N plasmid. (All curves except the red curve represent experiments done in the presence of 0.5 mM IPTG.) (B) Beta-galactosidase assays demonstrate that IPTG induces 402 (+/−7) fold increase in protein expression. LacZ was expressed from the pCA24N plasmid, either in the presence or the absence of IPTG, and levels of beta-galactosidase (Y-axis) were assayed according to standard procedures. The data summarized in this figure show that the Syn-IlvA protein is substantially less active than natural IlvA. The beta-galactosidase assays in panel B demonstrate that IPTG causes a 402 (+/−7) fold increase in protein expression. The results shown in panel A show that for the natural IlvA protein, this change in expression level has no effect on the rate of cell growth: Even low level expression in the absence of IPTG is sufficient for the natural protein to sustain robust growth. In contrast, cells that depend on Syn-IlvA grow slowly *even when expression is induced 400-fold.* Since cells relying on 400x levels of Syn-IlvA require significantly more time to grow than cells expressing 1x levels of the natural protein, the artificial protein is <1/400 as active as the natural protein. These experiments demonstrate that the artificial proteins are substantially less active in their ability to sustain cell growth. However, they do not indicate that the artificial proteins function by the same mechanism as the natural protein. Indeed, the reduced ability of the artificial proteins to sustain cell growth may be due to their functioning by a less effective mechanism.
(TIFF)

Figure S8 Structures of the 4 natural proteins compared with protein S-824, a *de novo* 4-helix bundle from a binary patterned library. Coordinates are from the PDB. (A) *M. jannaschii* SerB (1f5s). (B) *E. coli* GltA (1k3p). (C) The asymmetric unit of *E. coli* IlvA (1tdj). (D) *S. flexneri* Fes (3c87). (E) *De novo* protein S-824 (1p68).
(TIFF)
