# Supplementary material for: De Novo Designed Proteins from a Library of Artificial Sequences Function in Escherichia Coli and Enable Cell Growth
Source: PLoS One. 2011 Jan 4;6(1):e15364. doi: 10.1371/journal.pone.0015364 (PMC3014984; doi:10.1371/journal.pone.0015364)
Supplement: Text S1 — Supplementary materials and methods. (DOC) [file pone.0015364.s001.doc]

***Text S1. Supplementary materials and methods***

**Strains and plasmids.** Single-gene knockout strains of *E. coli* from the Keio collection (*1*) were provided by Wayne Patrick and Ichiro Matsumura at Emory University; or by Bryson Bennett, Joshua Rabinowitz, Natividad Ruiz, and Thomas J. Silhavy at Princeton University. The double knockout strains were constructed via standard techniques (*2,3*). The GFP-free variants of plasmids pCA24N, pCA24N-*lacZ*, pCA24N-*serB*, pCA24N-*ilvA*, pCA24N-*gltA*, and pCA24N-*fes* (*4*) were provided by the National Institute of Genetics, Shizuoka, Japan. The QUAD strain (*E. coli* K12 BW25113 Δ*fes* Δ*serB* Δ*gltA* Δ*ilvA*::kan) was constructed via standard techniques (*2, 3*). The expression vector pQLinkN was purchased from Addgene. In order to clone each artificial gene (*syn-fes-2*, *syn-* *serB-2*, *syn-gltA-1*, and *syn-ilvA-1*) into the multiple cloning site of pQLinkN, it was necessary to append 5’ BamHI and 3’ HindIII restriction sites via PCR amplification. This resulted in a 2-residue insertion of Gly-Ser immediately following the initiator Met for the sequences used in the quadruple knockout rescue experiment, as compared to those in the single-gene knockout experiments. Published methods were followed to combine the resulting plasmids in a systematic fashion, generating the co-expression constructs (*5*).

**Construction of expression vector pCA24NMAF2.** To express our library of artificial genes encoding *de novo* proteins, we reengineered GFP-free pCA24N (*4*) to insert 5’ BsrGI and 3’ NdeI sites; and to remove the original cloning site, the 6xHis tag, a linker, and the GFP removal scar sequence from the original vector. The new plasmid was named pCA24NMAF2. In this vector (as in the original pCA24N), protein expression is controlled by a T5 promoter and the lac operator.

**Verification of *de novo* sequences that enable cell viability.** Colonies that formed on the initial M9-glucose plates were purified by restreaking on fresh M9-glucose plates. Single colonies were grown in LB (30 μg ml^-1^ kan, 170 μg ml^-1^ cam). After 12 to 16 hours of growth, plasmid DNA was isolated and used to transform fresh heat-shock competent cells of the appropriate strain. Negative control plasmids (pCA24N-*lacZ* and pCA24NMAF2) and the appropriate positive control plasmid (pCA24N-*xxxX*) were also used in fresh transformations. The transformation outgrowth cultures were harvested, washed and resuspended in chilled M9 salts, and serial dilutions were spread on both M9-glucose and LB plates with the appropriate supplements. Appearance of a similar number of colonies on rich and minimal plates was taken as evidence that transfer of a *de novo* sequence is responsible for cell growth under selective conditions (Fig. 2C and Suppl. Fig. 1). Plasmid DNA from these hits was sequenced on both strands, and translated to amino acid sequences (Fig. 3 and Suppl. Fig. 2).

**Rescue of the quadruple-knockout auxotroph.** Individual expression constructs, co-expression constructs, or empty vector, all derivatives of pQLinkN, were used to transform electrocompetent QUAD cells. The transformation outgrowth cultures were harvested, washed and resuspended in chilled M9 salts, and serial dilutions were spread on both M9-glucose supplemented with 50 µM IPTG, 30 µg ml-1 kan, 150 µg ml-1 ampicillin (amp); and LB supplemented with 30 µg ml-1 kan, 150 µg ml-1 amp. Appearance of a similar number of colonies on rich and minimal plates after incubation at 33°C was taken as evidence that transfer of *de novo* sequences is responsible for cell growth under selective conditions.

**Preparation of clarified cell lysates and assay for iron.** Single colonies from freshly transformed cells were used to inoculate LB (30 μg ml^-1^ kan, 170 μg ml^-1^ cam). After overnight growth at 37°C, cultures were diluted 10-fold into LB (30 μg ml^-1^ kan, 34 μg ml^-1^ cam.) Cultures were grown to A_600 nm_  of ~0.4 and expression was induced with 0.5 mM IPTG. Cultures were grown for another 6.5 hours and then centrifuged. Cell pellets were frozen at -20°C. Cells were thawed on ice, resuspended, and disrupted in BugBuster HT Protein Extraction Reagent (Novagen). Following centrifugation, supernatants were diluted 2-fold with 50 mM Tris, 200 mM NaCl, pH 8.1, incubated for 10 min. at 100°C, and centrifuged. Pellets were assayed for iron using inductively coupled plasma spectrometric analysis (ICP - Alliance Technologies) (Suppl. Fig. 6).

**References for supporting material**

1. Baba T, Ara T, Hasegawa M, Takai Y, Okumura Y, et al. (2006) Construction of

*Escherichia coli* K-12 in-frame, single-gene knockout mutants: The Keio collection. Mol

Syst Biol 2: 2006.0008.

# 2. Datsenko KA, Wanner BL (2000) One-step inactivation of chromosomal genes in **Escherichia coli** K-12 using PCR products Proc Natl Acad Sci USA 97, 6640-6645

3. Sambrook J, Russell DW (2001) Molecular Cloning: A Laboratory Manual (Cold Spring Harbor Lab. Press, Cold Spring Harbor, NY, 3^rd^ ed.).

4. Kitagawa M, Ara T, Arifuzzaman M, Ioka-Nakamichi T, Inamoto E, et al. (2005) Complete set of ORF clones of *Escherichia coli* ASKA library (A complete Set of *E. coli* K-12 ORF Archive): Unique resources for biological research. DNA Res 12: 291-299.

5. Scheich C, Kummel D, Soumailakakis , Heinemann U, Bussow K (2007) Vectors for co-expression of an unrestricted number of proteins. Nucleic Acids Res 35: e43.

6. Bradley LH, Kleiner RE, Wang AF, Hecht MH, Wood DW (2005) An intein-based genetic selection allows the construction of a high-quality library of binary patterned *de novo* protein sequences. Protein Eng Des Sel 18: 201-207.
