# Supplementary figures and images for: De Novo Designed Proteins from a Library of Artificial Sequences Function in Escherichia Coli and Enable Cell Growth
Source: PLoS One. 2011 Jan 4;6(1):e15364. doi: 10.1371/journal.pone.0015364 (PMC3014984; doi:10.1371/journal.pone.0015364)

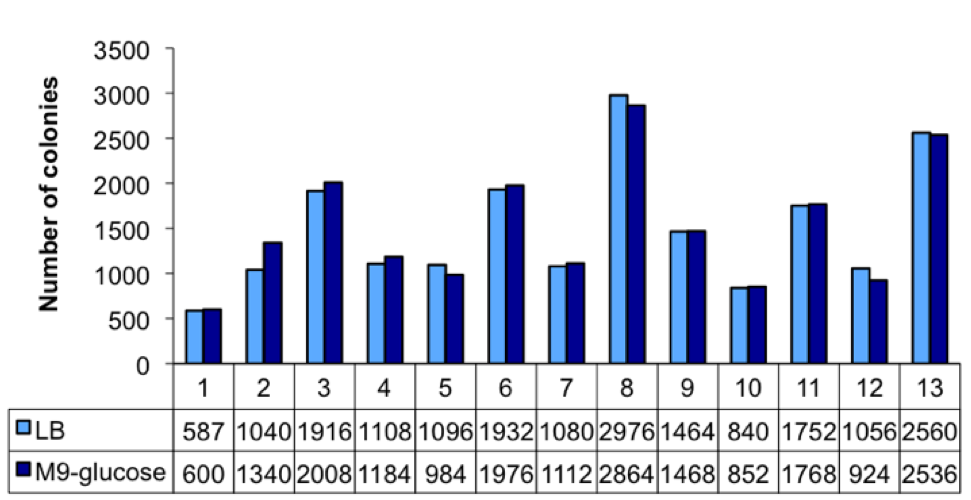

Supplement: Figure S1 — Reconfirmation of hits. Colony counts for re-transformation of ΔilvA cells with several different hits. The histogram summarizes 13 experiments of the type shown in figure 2C. The number of colonies on M9-glucose minimal media (dark blue) is similar to the number on LB rich media (light blue). (TIFF) [file pone.0015364.s002.tif]

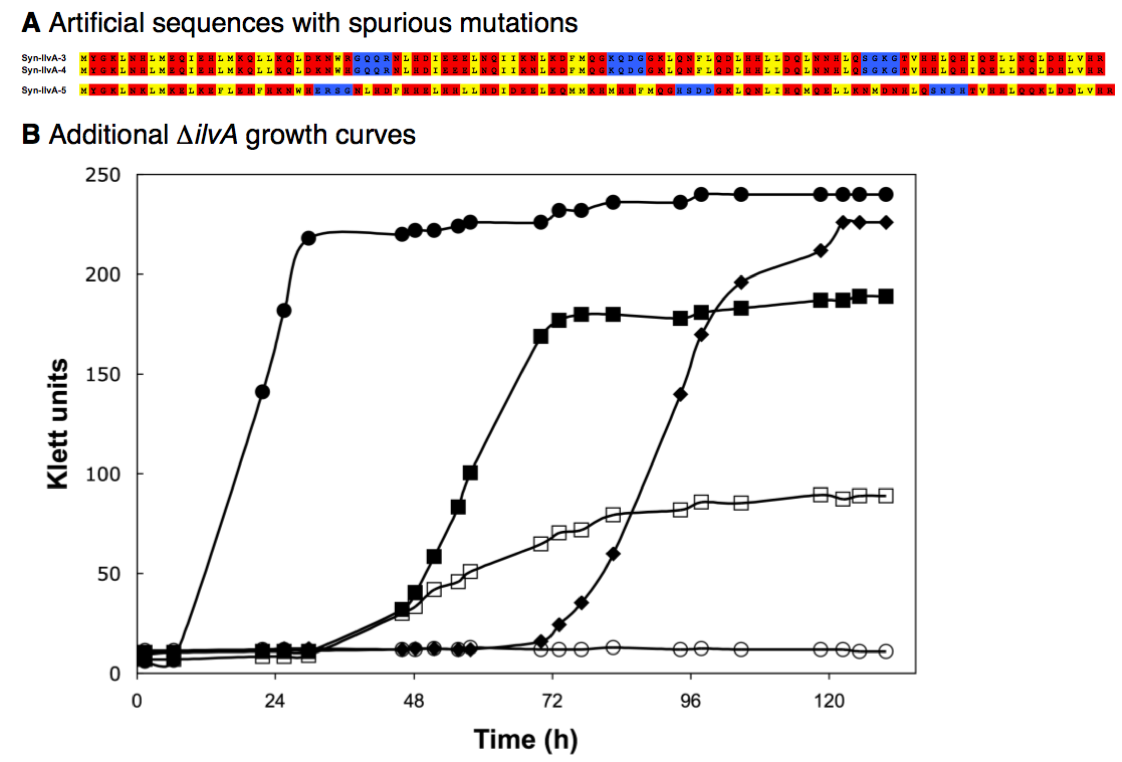

Supplement: Figure S2 — De novo sequences with spurious mutations and corresponding ΔilvA growth curves. (A) Novel sequences with spurious mutations. Red indicates polar residues, yellow indicates nonpolar residues, and turns are in blue. Syn-IlvA-3 and Syn-IlvA-4 are 105-residue proteins differing by a single residue at position 28. Both exhibit a 4 amino acid insertion into helix 1, and a 1 amino acid deletion from helix 4. Syn-IlvA-5 is a 106-residue protein, with an 11 amino acid insertion into the second helix and a 7 amino acid deletion from helix 4. These spurious mutations occur at the DNA level and are due either to errors in oligonucleotide synthesis or rare instances of the mis-assembly of library insert building blocks. As long as such spurious mutations do not affect frame or introduce stop codons, the mutated segments survive preselection, and as shown by our results, can contribute to the synthesis of productive sequences. (B) Growth curves for the ΔilvA strain expressing designed artificial proteins with spurious mutations. The bottom curve shows that the negative control expressing LacZ (open circles) does not enable growth. In contrast, the positive control expressing IlvA (closed circles) grows well. Like the novel sequences that adhere precisely to the design, the designed proteins with spurious mutations enable growth that is well above background. Growth curves correspond to the sequences in A as follows: Syn-IlvA-3, closed squares; Syn-IlvA-4, open squares; and Syn-IlvA-5, closed diamonds. (TIFF) [file pone.0015364.s003.tif]

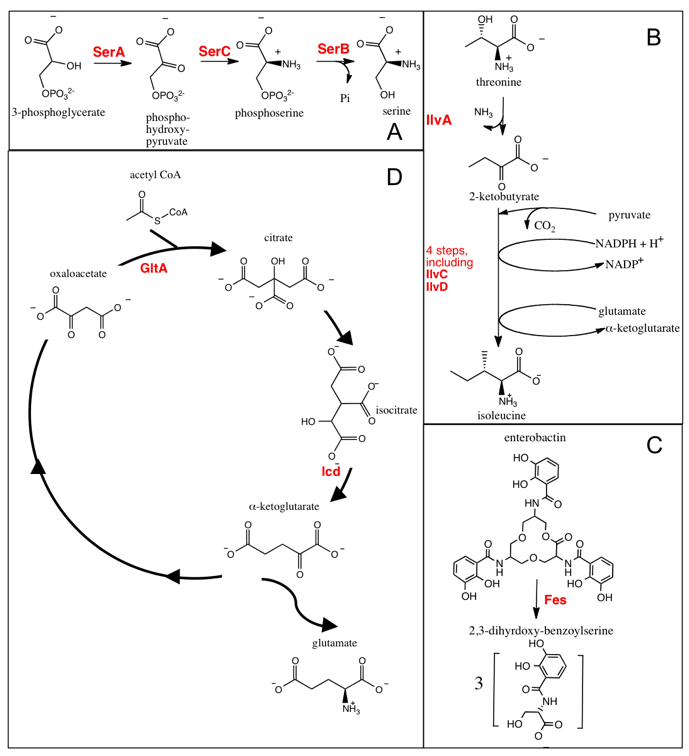

Supplement: Figure S3 — Reactions and pathways of the deleted proteins. (A) serB encodes phosphoserine phosphatase, responsible for the final step in serine biosynthesis. (B) ilvA encodes biosynthetic threonine deaminase, which catalyzes the first step in the production of isoleucine from threonine. (C) fes encodes enterobactin esterase, which cleaves iron-bound enterobactin. (D) gltA encodes citrate synthase, which catalyzes an early step in glutamate biosynthesis. (TIFF) [file pone.0015364.s004.tif]

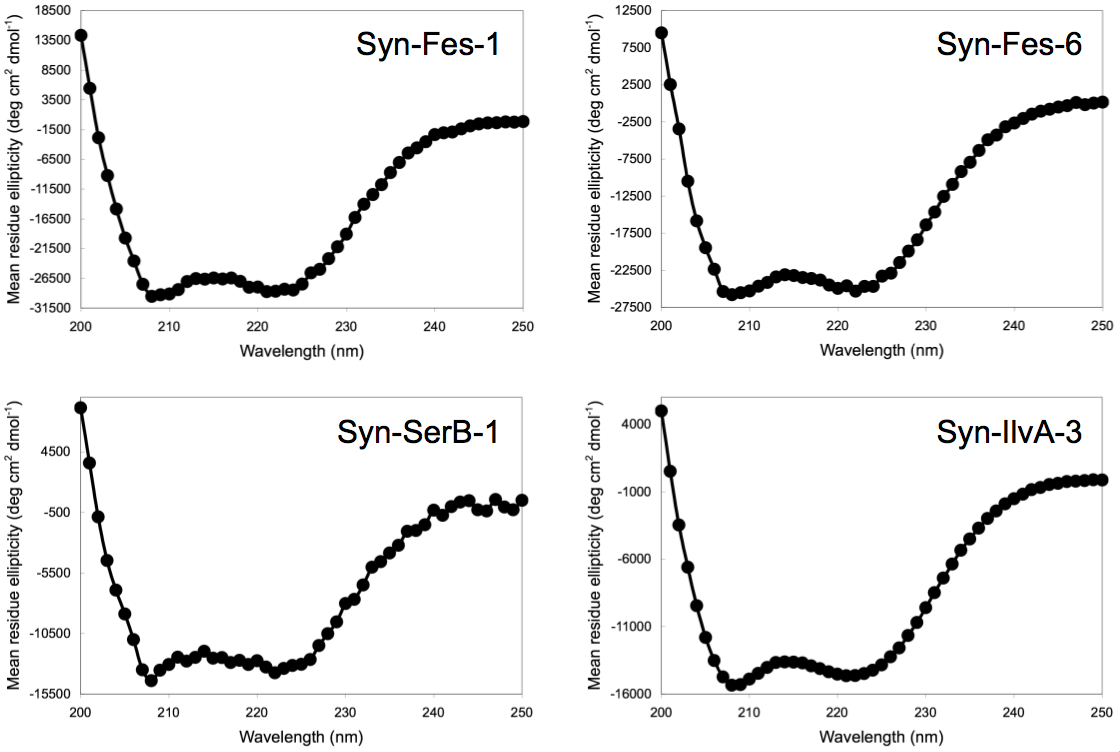

Supplement: Figure S4 — Circular dichroism spectra. CD spectra demonstrate that purified de novo proteins fold into alpha-helical structures. Cultures of E. coli were grown to an OD600 of approximately 0.6 and induced with IPTG. Protein was extracted from the cells by repeated cycles of freezing and thawing. Purification of the Syn proteins to 95% (coommaise-stained SDS-PAGE) was achieved by cation exchange chromatography. Proteins were dialyzed into phosphate buffer for CD analysis. (TIFF) [file pone.0015364.s005.tif]

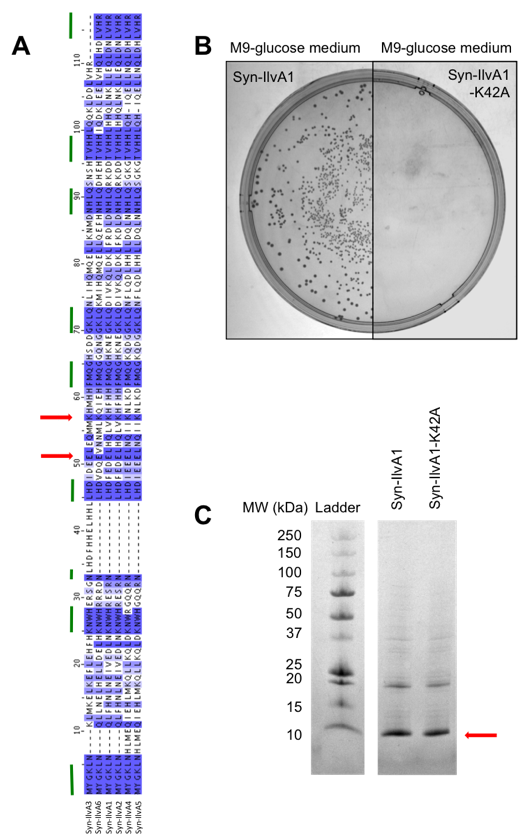

Supplement: Figure S5 — Mutation of Lys42 to Ala destroys the ability of a synthetic protein to rescue ΔilvA. (A) Sequence alignment of the six synthetic proteins that rescue ΔilvA. Two charged residues are conserved, E and K (red arrows). Green lines mark regions that are conserved in the designed template. (B) Transformation of ΔilvA cells with Syn-IlvA-1 and Syn-IlvA-1-K42A. Cells expressing Syn-IlvA-1 grow on M9-glucose, but cells expressing Syn-IlvA-1-K42A do not. (C) SDS-PAGE of protein levels after induction with IPTG. Levels of Syn-IlvA-1-K42A are approximately equivalent to those of Syn-IlvA-1. Red arrow marks the band corresponding to the synthetic protein. (TIFF) [file pone.0015364.s006.tif]

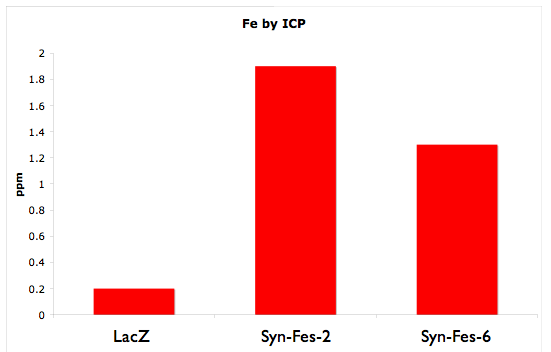

Supplement: Figure S6 — Assay for iron using inductively coupled plasma (ICP) spectrometry. The Δfes strain was transformed with a plasmid directing the high level expression of either LacZ or one of the artificial sequences that rescued the fes deletion. Cells were grown in LB, harvested, and tested for iron accumulation. The artificial proteins enable the accumulation of 6-10 fold more iron than the control. (TIFF) [file pone.0015364.s007.tif]

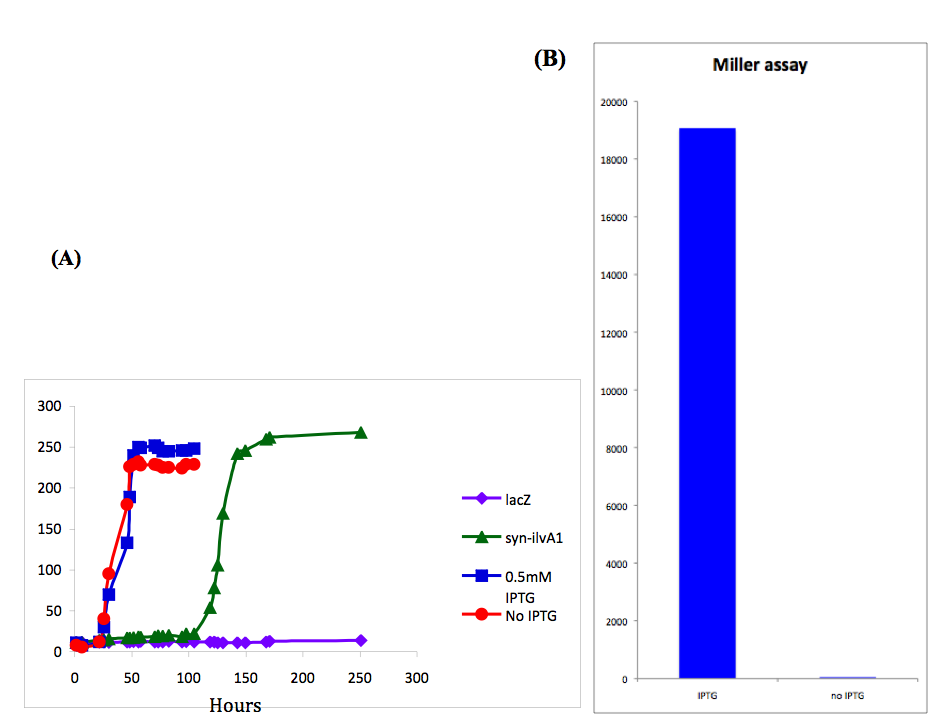

Supplement: Figure S7 — De novo proteins are considerably less active than the natural proteins they replace. (A) Growth on minimal media of ΔilvA cells expressing different sequences. Cells expressing the natural IlvA protein grow well either in the presence (Blue) or absence (Red) of IPTG. In contrast, cells expressing Syn-IlvA-1 grow significantly more slowly, even in the presence of IPTG (Green). [These cells do not grow in the absence of IPTG.] Control cells expressing LacZ fail to grow even in the presence of IPTG (Purple). All proteins were expressed off the pCA24N plasmid. (All curves except the red curve represent experiments done in the presence of 0.5 mM IPTG.) (B) Beta-galactosidase assays demonstrate that IPTG induces 402 (+/−7) fold increase in protein expression. LacZ was expressed from the pCA24N plasmid, either in the presence or the absence of IPTG, and levels of beta-galactosidase (Y-axis) were assayed according to standard procedures. The data summarized in this figure show that the Syn-IlvA protein is substantially less active than natural IlvA. The beta-galactosidase assays in panel B demonstrate that IPTG causes a 402 (+/−7) fold increase in protein expression. The results shown in panel A show that for the natural IlvA protein, this change in expression level has no effect on the rate of cell growth: Even low level expression in the absence of IPTG is sufficient for the natural protein to sustain robust growth. In contrast, cells that depend on Syn-IlvA grow slowly even when expression is induced 400-fold. Since cells relying on 400x levels of Syn-IlvA require significantly more time to grow than cells expressing 1x levels of the natural protein, the artificial protein is <1/400 as active as the natural protein. These experiments demonstrate that the artificial proteins are substantially less active in their ability to sustain cell growth. However, they do not indicate that the artificial proteins function by the same mechanism as the natural protein. Indeed, the re [file pone.0015364.s008.tif]

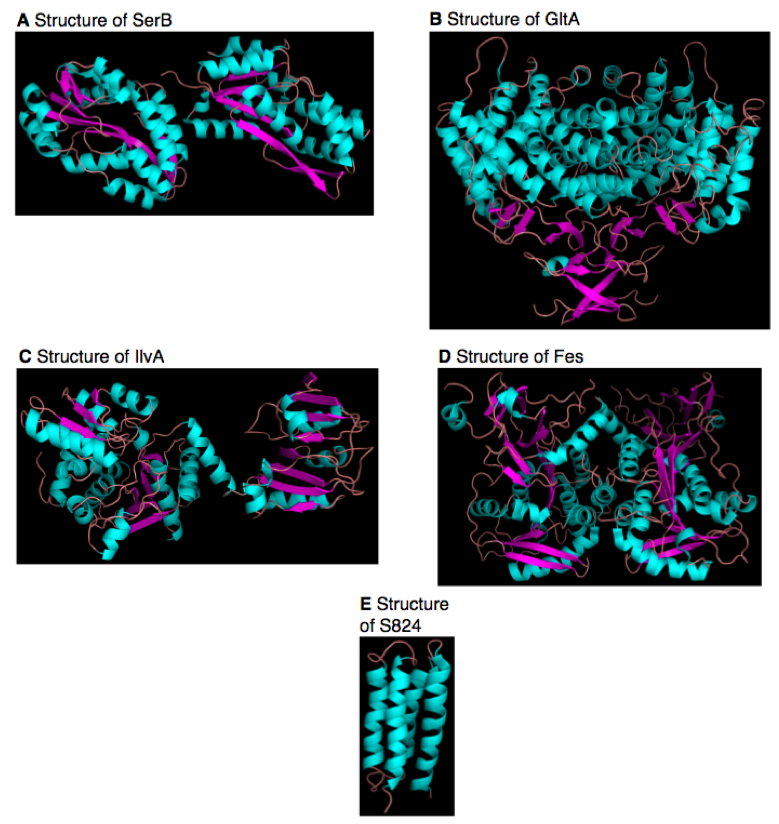

Supplement: Figure S8 — Structures of the 4 natural proteins compared with protein S-824, a de novo 4-helix bundle from a binary patterned library. Coordinates are from the PDB. (A) M. jannaschii SerB (1f5s). (B) E. coli GltA (1k3p). (C) The asymmetric unit of E. coli IlvA (1tdj). (D) S. flexneri Fes (3c87). (E) De novo protein S-824 (1p68). (TIFF) [file pone.0015364.s009.tif]
